# A novel diagnostic model for differentiation of lung metastasis from primary lung cancer in patients with colorectal cancer

**DOI:** 10.3389/fonc.2022.1017618

**Published:** 2022-10-24

**Authors:** Rui Guo, Shi Yan, Fei Wang, Hua Su, Qing Xie, Wei Zhao, Zhi Yang, Nan Li, Jiangyuan Yu

**Affiliations:** ^1^Key Laboratory of Carcinogenesis and Translational Research (Ministry of Education, Beijing), National Medical Products Administration (NPMA) Key Laboratory for Research and Evaluation of Radiopharmaceuticals (National Medical Products Administration), Department of Nuclear Medicine, Peking University Cancer Hospital & Institute, Beijing, China; ^2^Key Laboratory of Carcinogenesis and Translational Research (Ministry of Education/Beijing), Department of Thoracic Surgery II, Peking University Cancer Hospital and Institute, Beijing, China

**Keywords:** PET/CT, lung lesion, primary lung cancer, lung metastasis, colorectal cancer

## Abstract

**Objective:**

This study aimed to evaluate the ^18^F-FDG PET/CT in differentiating lung metastasis(LM) from primary lung cancer(LC) in patients with colorectal cancer (CRC).

**Methods:**

A total of 120 CRC patients (80 male, 40 female) who underwent ^18^F-FDG PET/CT were included. The diagnosis of primary lung cancer or lung metastasis was based on histopathology The patients were divided into a training cohort and a validation cohort randomized 1:1. Independent risk factors were extracted through the clinical information and ^18^F-FDG PET/CT imaging characteristics of patients in the validation cohort, and then a diagnostic model was constructed and a nomograms was made. ROC curve, calibration curve, cutoff, sensitivity, specificity, and accuracy were used to evaluate the prediction performance of the diagnostic model.

**Results:**

One hundred and twenty Indeterminate lung lesions (ILLs) (77 lung metastasis, 43 primary lung cancer) were analyzed. No significant difference in clinical characteristics and imaging features between the training and the validation cohorts (*P* > 0. 05). Using uni-/multivariate analysis, pleural tags and contour were identified as independent predictors. These independent predictors were used to establish a diagnostic model with areas under the receiver operating characteristic curves (AUCs) of 0.92 and 0.89 in the primary and validation cohorts, respectively. The accuracy rate of the diagnostic model for differentiating LM from LC were higher than that of subjective diagnosis (*P* < 0.05).

**Conclusions:**

Pleural tags and contour were identified as independent predictors. The diagnostic model of ILLs in patients with CRC could help differentiate between LM and LC.

## Introduction

Colorectal cancer (CRC) is the third most commonly diagnosed malignancy and the fourth leading cause of cancer-related deaths in the world (1). In patients with CRC, the lung is the second most common site of metastasis, and pulmonary metastasis has been detected in 10%-22% of all CRC patients (2, 3). Indeterminate lung lesions (ILLs) includes a malignancy (either lung metastasis or primary lung cancer) or a benign. The definition of the nature of ILLs is important for accurate staging and decision-making further diagnostic workup and therapeutic strategy. In addition, surgical strategies for treating primary lung cancer (LC) and lung metastasis (LM) are very different. The treatment option for LM is minimally invasive surgical resection in order to preserve as much healthy lung parenchyma as possible in case repeated surgery is required. In contrast, complete surgical resection with lobotomy and mediastinal lymph node dissection is the gold standard for LC (4).

It is sometimes difficult to determine whether an ILL is a primary LC or a LM. There are reports that the incidence of LM is higher in patients with CRC, the incidence of primary lung cancer in CRC patients was reported to be 2.8%-23% (5–7). There are also reports that primary LC is similar to LM (5–7). Bronchoscopy and/or transthoracic fine-needle aspiration biopsy can help distinguish primary LC from LM before surgical. However, sometimes these strategies are difficult and dangerous, especially for those with small lesions or its deep parenchymal location. Therefore, the clinical and imaging characteristics of the patients, which are of great importance for a physician to make judgment whether it is an primary LC or LM, especially when a patient faces those who can’t accomplish biopsy, because treatment strategies may be completely different. However, studies using clinical and radiological findings to discriminate LM from LC are limited (8). Imaging characteristics of ILLs can be used as a noninvasive alternative to determine whether it is primary LC or LM. ^18^F-fluorodeoxyglucose(FDG)-positron emission tomography/computed tomography (PET/CT) is an accurate and non-invasive imaging method for evaluating pulmonary nodules and mass lesions (9–11). This noninvasive functional imaging test takes advantage of the observed increase in glucose metabolism in malignant cells and is gaining acceptance in oncology for tumor diagnosis. Over the past decades, ^18^F-FDG-PET/CT has been identified as the main component of the management of cancer patients (12).PET images are mainly analyzed by vision, but quantitative analysis can also be performed. Tissue glucose utilization can be assessed semi-quantitatively by standard uptake value (SUVmax) in the lesion, providing an observer-independent and repeatability measurement (13, 14). The significance of ^18^F-FDG PET/CT in the diagnosis or prognosis of ILLs has been reported previously (15, 16). Unfortunately, few studies provide the clinical features and ^18^F-FDG PET/CT features to discriminate LC from LM in CRCs patients. Therefore, the purpose of this study is attempt to establish a diagnostic model to distinguish primary LC and LM of CRC patients.

## Materials and methods

### Patients

**Figure 1:**
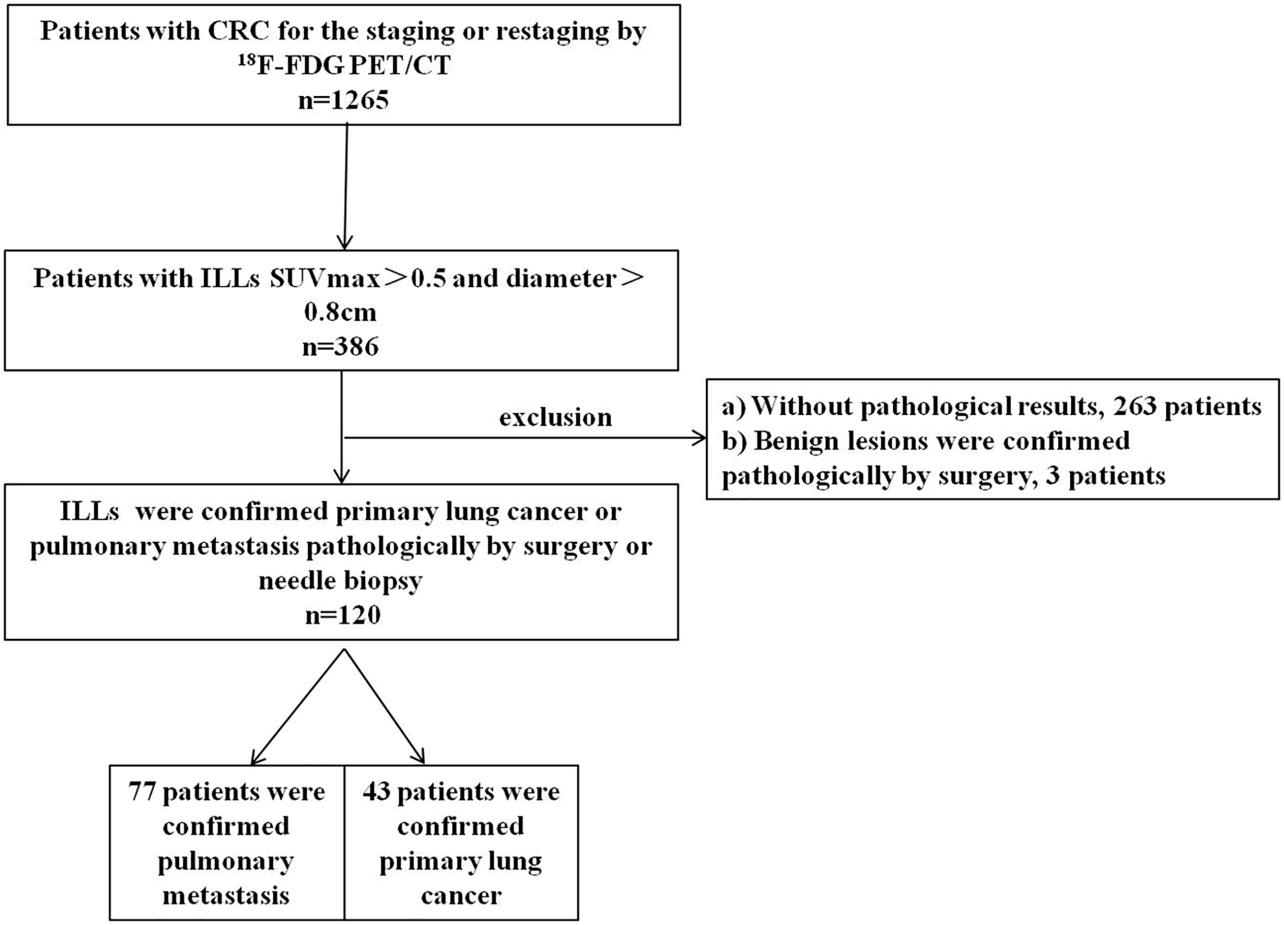
Flowchart of patient enrollment.

We retrospectively reviewed CRC patients underwent ^18^F-FDG PET/CT from January 2012 to December 2018 at our hospital. The inclusion criteria included the following: (1) CRC was diagnosed by pathology; (2) pulmonary lesions SUVmax>0.5 and diameter>0.8cm (17); (3) pulmonary lesions were pathologically confirmed by surgery or needle biopsy. A total of 123 patients were eligible, of which 3 benign patients were excluded because the sample size was too small. The complete patient enrollment process is shown in **Figure 1**. Finally, 120 CRC patients(40 female and 80 male) with a mean age of 62.68 ± 9.70 years (range: 35–85 years) were enrolled in this study. Since this study is retrospective, national laws require neither institutional review board approval nor informed consent.

### PET/CT examination

All patients underwent whole-body PET/CT acquisition 60 ± 10 min after injection ^18^F-FDG by 3.7 MBq/kg. Prior to FDG injection, all patients fasted for at least 6 h. In all cases, the serum glucose concentration met the institutional requirement (≤120 mg/dL).

PET/CT scans were conducted by a Siemens Biograph mCT Flow 64 scanner (Siemens, Erlangen, Germany) or Gemini TF scanner (Philips Medical Systems, The Netherlands) which covered the length from the top of skull to the mid-thigh. A low-dose CT scan (120 kV, 35 mA, slice 3 mm) was first performed, and PET acquisition speed was 1.5 mm/s (slice 3 mm, filter: Gaussian, FWHM: 5 mm) or scanning at a total of 9–10 bed positions, a 90-s acquisition time for each bed position. A Siemens Biograph mCT Flow 64 scanner PET images were reconstructed using a three-dimensional iterative reconstruction with the time-of-flight algorithm, and the low-dose CT scans were acquired in CARE Dose 4D mode. A Gemini TF scanner PET reconstruction parameters included use of 3D model, and use of ordered-subcohorts expectation maximization(OSEM) method (two iterations, four subcohorts, 128×128 pixels of 5.15 mm). Attenuation corrections of the PET images were performed using data from CT imaging.

### Image analysis

A Siemens workstation (MultiModality Workplace, Siemens, Germany) was used for post processing. Two experienced nuclear medicine practitioners reviewed and analyzed the ^18^F-FDG PET/CT independently, and any inconsistencies were resolved by consensus.

Volumes of interest (VOIs) were manually drawn for each lesion and the SUVmax values were automatically calculated. The axial, coronal, and sagittal ^18^F-FDG PET/CT images were qualitatively analyzed by nuclear medicine physicians. The clinical characters included patients: age, gender, smoking history, index tumor location, index tumor stage(TNM, The pathologic staging system was based on the 8th edition of the Union for International Cancer Control/American Joint Committee on Cancer (UICC/AJCC)), whether exist extrapulmonary metastasis or vascular tumor thrombus, carcinoembryonic antigen (CEA) level when ILLs were detected, disease free interval (DFI) considered as the time between the diagnosis of CRC and the lung lesions by ^18^F-FDG PET/CT detection. Radiological characteristics of the lung lesions in terms of size, SUVmax, contour, location (left or right, upper,middle or lower, central or peripheral), margin(smooth, lobulated, or spiculated), presence of ground-glass opacity (GGO),air bronchogram, cavitation, pleural tags. Lesios were classified as smooth, lobulated, or spiculated based on margin characteristics. An air bronchogram was defined as a gas-filled bronchus surrounded by abnormal lung parenchyma. Pleural tags were defined as linear strands that extended between nodule surface and adjacent pleural surface. In addition,we compared with the uptake intensity in the mediastinum, the intensity of FDG uptake was also visually analyzed to try to further optimize the characterization of ILL.

### Statistical analysis

Statistical analysis were conducted by using IBM SPSS (Version 22.0; IBM Corp., New York, USA) and R package 3.6.2 (R Foundation for Statistical Computing, Vienna, Austria). A two-sided P less than 0.05 indicated statistical significance.

The measurement data were described as means ± standard deviation, and analyzed using Student’s t-test or Mann-Whitney U-test. The enumeration data were described as numbers and percentages, and analyzed using the chi-square test. Multivariate analysis for predicting lung cancer and lung metastasis was performed using logistic regression by incorporating variables with P < 0.05 in univariate analysis. The nomogram and calibration curve were drawn for the multivariate model. The receiver operating characteristic (ROC) curve was used to assess the diagnostic performance. The area under the curve (AUC) was calculated, and the cutoff was determined using the maximum Youden’s method. Sensitivity, specificity, positive predictive value (PPV), negative predictive value (NPV) and accuracy were calculated. The accuracy between the model and nuclear medicine physicians were compared using McNemar test. Inter-reader agreement for diagnosis results was assessed by kappa of agreement. A value of kappa lower than 0.20 was interpreted as poor agreement, 0.41–0.60 as moderate, 0.61–0.80 as substantial, and 0.81–1as almost perfect agreement.

## Results

### Patient and ILLS characteristics

After exclusion of ILLs for those SUVmax <0.5,and diameter<0.8cm,and no pathological findings, 123 lesions with pathological results remained for analysis. Among 123 lesions, 3 benign lesions were excluded in the final. The clinical information of the 120patients are summarized in **Table 1**, 80 males and 40 females, the mean age 62.68 ± 9.70 years old, 77 lung metastasis (64.2%) and 43 primary lung cancer (35.8%), respectively. Simultaneous lung metastasis was observed in 16 patients, and 5 patients with synchronous multiple primary carcinomas. ILLs characteristics are presented in **Table 1**. There was no statistically significant difference in the clinical characteristics and ILLs imaging of the patients between the training cohort and the validation cohort (*P* > 0.05, **Table 1**).

**Table 1:** Clinical characteristics of patients and CRCs.

Characteristic	Total	Training cohort	Validation cohort	*P* value
No. of patients	120	60	60	
Sex (male/female)		42/18	38/22	0.439
Age (years)		62.93 ± 9.30	62.42 ± 10.15	0.772
Pathology				0.849
LM	77	39	38	
LC	43	21	22	
History of smoking	51	24	27	0.58
Index tumor location				0.369
Right colon	17	6	11	
Left colon	29	14	15	
Rectum	74	40	34	
Index tumor stage				0.733
I	23	10	13	
II	44	24	20	
III	45	23	22	
IV	8	3	5	
Extrapulmonary metastasis				0.432
Exist	17	7	10	
None	103	53	50	
Vascular tumor thrombus				>0.999
Exist	34	17	17	
None	86	43	43	
CEA				0.709
Rise	48	25	23	
Normal	72	35	37	
DFI		26.75 ± 29.63	35.10 ± 33.28	0.153
Size(cm)		2.02 ± 1.41	1.91 ± 0.98	0.614
SUVmax		6.34 ± 4.34	6.46 ± 4.29	0.873
≤Mediastinum	18	7	11	0.306
>Mediastinum	102	53	49	
Contour				0.444
Circular	42	23	19	
Non-circular	78	37	41	
Location				>0.05
Central	24	12	12	
Peripheral	96	48	48	
Right lung	69	37	32	
Left lung	51	23	28	
Upper lobe	57	31	26	
Middle lobe	10	7	3	
Lower lobe	53	22	31	
Margin				>0.05
Smooth	38	19	19	
Lobulated	94	47	47	
Spiculated	39	18	21	
Air bronchogram	5	2	3	>0.999
Pleural tags	57	26	31	0.361
Cavitation	14	7	7	
GGO	11	6	5	0.752

### Uni-/multivariate analysis in the training cohort

The training cohort was divided into two groups, one for LM and one for LC. Except for age, the difference in clinical characteristics between the two groups was not statistically significant in univariate analysis (**Supplementary Table S1**). ^18^F-FDG PET/CT features of ILLs were compared between LM and LC groups (**Supplementary Table S2**). The mean size of ILLs was significantly greater in the LC group (2.28 ± 1.12cm) than in the LM group (1.71 ± 0.84cm) (*P*<0.05). The presence of ILLs with contour, margin(smooth, lobulated, or spiculated), an air bronchogram, pleural tags,and GGO were significantly between LC and LM group (*P*< 0.005). On multivariate analysis including these 9 factors as variables of interest, contour, pleural tags were identified as significant independent factors for discriminating primary LC from LM (**Table 2**). **Table 3** shows the logistic regression models for differentiating LM from LC of CRC patients. The results revealed that ILLs with pleural tags had a higher rate of diagnosing LC(odds ratio (OR) =28.504; 95% confidence interval (CI): 3.966-204.865) and ILLs with circular contour had a higher rate of diagnosing LM(odds ratio (OR) =0.059; 95% confidence interval (CI): 0.006-0.581).

**Table 2:** The results of multivariate analysis in the training cohort.

Variable			Univariate	Multivariate
	LM(n=39)	LC(n=21)	*P* value	*P* value
Age (years)	59.44 ± 10.39	67.95 ± 7.03	0.001	
Size(cm)	1.71 ± 0.84	2.28 ± 1.12	0.029	
Contour				
Circular	18(46.2%)	1(4.0%)	0.001	0.015
Margin			<0.05	
Smooth	19(48.7%)	0(0%)		
Lobulated	27(69.2%)	20(95.2%)		
Spiculated	7(17.9%)	14(66.7%)		
Air bronchogram	0(0%)	3(14.3%)	0.039	
Pleural tags	13(33.3%)	18(85.7%)	<0.001	0.001
GGO	0(0%)	5(23.8%)	0.004	

**Table 3:** Multivariate analysis results of diagnostic model for differentiation LM from LC in training cohort.

Differentiation of ILLs	Characteristics of ILLs	B	OR	95%CI	P
LM vs LC	Pleural tags	3.35	28.504	3.966-204.865	0.001
	Contour	-2.838	0.059	0.006-0.581	0.002

B, regression coefficient; OR, odds ratio.

### Construction of the diagnostic model

**Figure 2:**
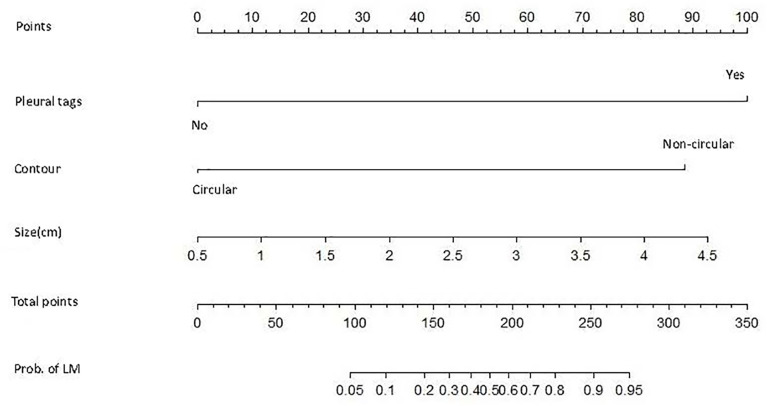
The nomograms for the diagnostic model of CRC patients.

**Figure 3:**
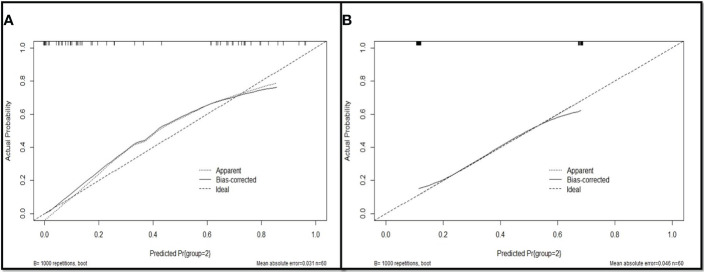
Evaluation of the nomogram for the diagnostic model for differentiating LM from LC of CRC patients. **(A)** The calibration curves of training cohort; **(B)** The calibration curves of validation cohort.

**Figure 4:**
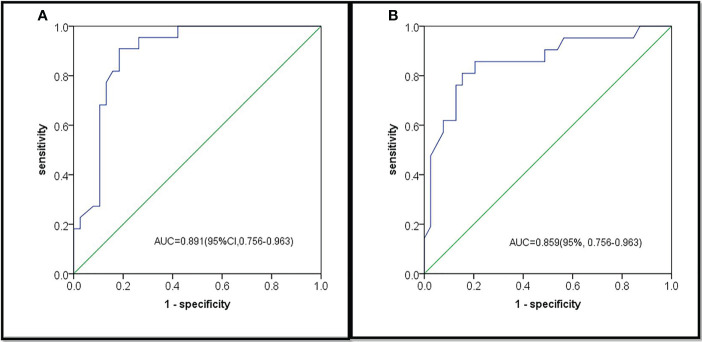
Receiver operating characteristics (ROC) curves analysis of diagnostic model for differentiating LM from LC. **(A)**The AUC of diagnostic model for diagnosing LM and LC was 0.891 in the training cohort; **(B)** The AUC of diagnostic model for diagnosing LM and LC was 0.859 in the validation cohort.

**Figure 5:**
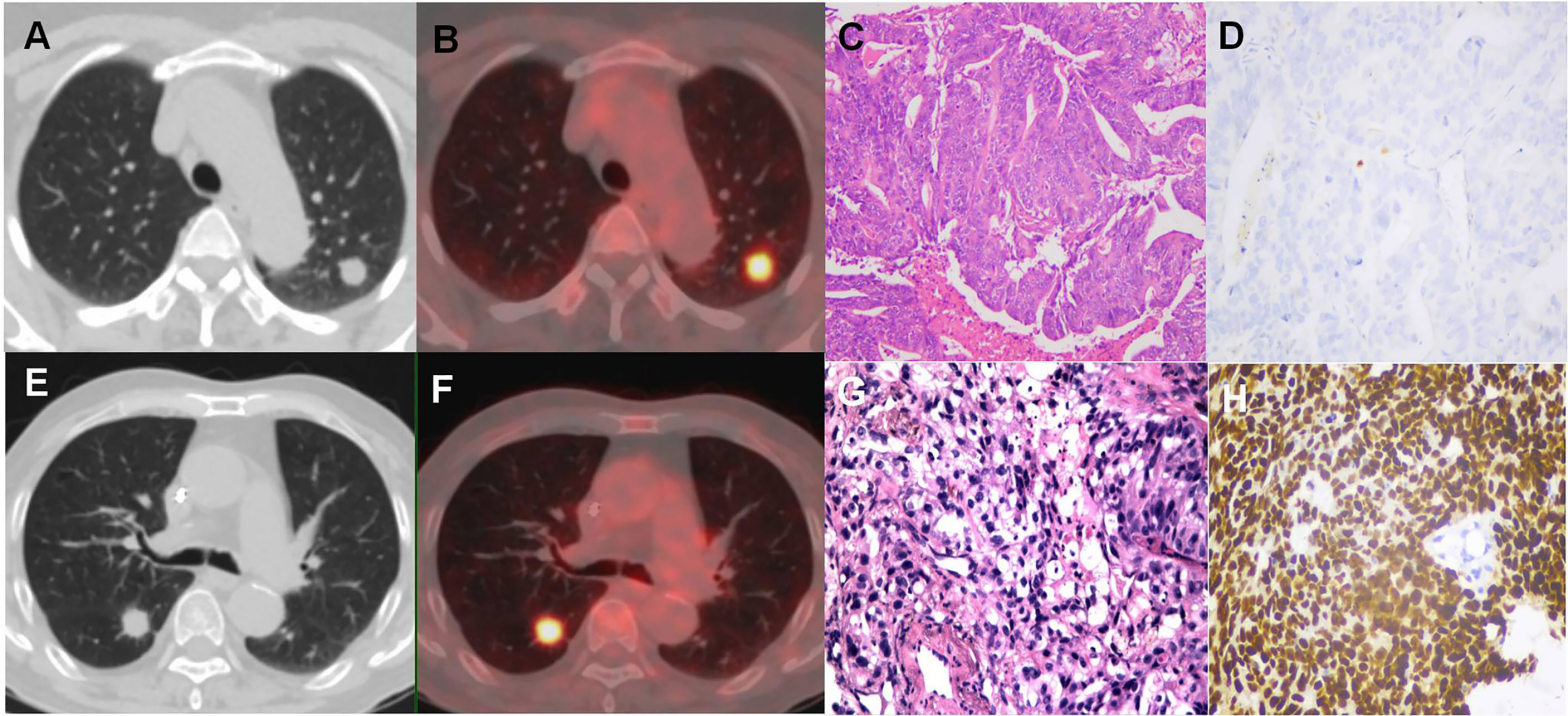
^18^F-FDG PET/CT findings of LM and LC of CRC patients. **(A, B)** 49y female was diagnosis sigmoid colon cancer. 18F-FDG PET/CT showed a circular soft tissue nodule in upper lobe of the left lung, which with smooth margin and high FDG uptake on CT and integrated PET-CT. The nodule was histopathologically confirmed to be LM; **(C, D)** The hematoxylin-eosin staining (200x) showed that the adenocarcinoma cells grew infiltratively with clear cytoplasm. Immunohistochemistry was negative for TTF-1; **(E, F)** 71y male was diagnosis sigmoid colon cancer. ^18^F-FDG PET/CT showed a circular soft tissue nodule of high FDG uptake in inferior lobe of right lung, which with spiculated margin and pleural tags on CT and integrated PET/CT.; The nodule was histopathologically confirmed to be LC; **(G, H)** The hematoxylin-eosin staining (200x) showed that adenocarcinoma cells are confluent, cribriform infiltrative, and necrotic tissue can be seen. Immunohistochemistry showed TTF-1 positive.

Based on multivariate analysis results the diagnostic model were used to construct for discriminating primary LC from LM with CRC. The differential diagnosis prediction model was presented as: Y1 = 3.35* pleural tags -2.838* contour +0.844*size. The area under ROC curve of logistic differentiation model of training cohort was 0.89 (95%CI,0.808-0.973), Y1≥3, which was diagnosed as lung cancer, and Y1<3, which was diagnosed as lung metastasis. The area under ROC curve of validation cohort was 0.86 (95%CI, 0.756-0.963). Based on the predictive factors in the multivariable analysis, nomogram was constructed to differentiate LM from LC of CRCs (**Figure 2**). The calibration curves of differentiate LM from LC of CRCs suggested a good agreement between the training cohort and validation cohort (**Figure 3**). The diagnostic model showed an AUC of 0.891(95%CI, 0.808-0.973) and an accuracy of 85% in the training cohort (**Figure 4A**), and an AUC of 0.859(95%CI,0.756-0.963) and an accuracy of 80.0% in the validation cohort (**Figure 4B**). Detailed information on the performance of the diagnostic models for differentiating LM from LC of ILLs with CRC is shown in **Table 3**. A typical case is shown in **Figure 5**.

### Advantage of the diagnostic model

**Figure 6:**
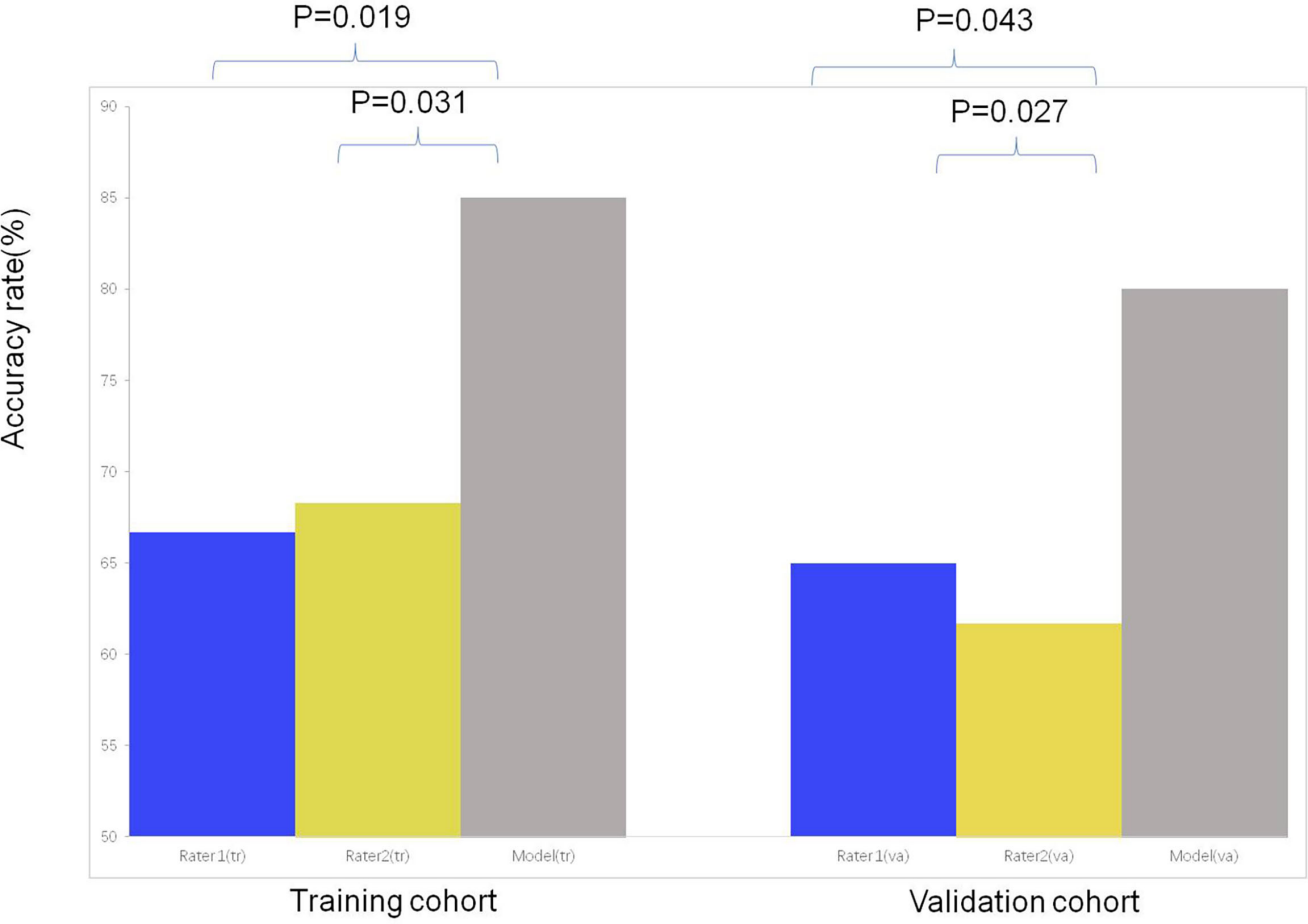
The comparison of the accuracy among subjective evaluation and subjective evaluation with the assistance of diagnostic model for differentiating LC from LM of CRC.

Inter-observer agreement for diagnosis LM with LC was near perfect (kappa=0.850).In the training and validation cohort, both nuclear medicine physicians were less effective at differentiating LM and LC(both P<0.05) than diagnostic model. The sensitivity, specificity, PPV, NPV and accuracy of diagnostic model and experts for diagnosis in differentiating LM from LC of ILLs are shown in **Table 4** and **Supplementary Tables S3, S4**. In the training and validation cohort, accuracy rates were significant by using diagnostic model than experts (both P <0.05) (**Figure 6**).

**Table 4:** Comparison between subjective evaluation and diagnostic model diagnosis.

	Sensitivity, %	Specificity, %	PPV, %	NPV, %	Accuracy, %
Training cohort					
Diagnostic model	90.5	82.1	73.1	94.1	85.0
R1	81.0	59.0	51.5	85.2	66.7
R2	76.2	64.1	48.5	83.3	68.3
Validation cohort					
Diagnostic model	81.8	78.9	69.2	88.2	80.0
R1	72.7	68.2	50.0	76.7	63.3
R2	68.2	68.2	48.4	75.9	61.7

R, rater; PPV, positive predictive value; NPV, negative predictive value.

## Discussion

Lung is generally considered to be one of the most common organs for secondary primary tumors (18, 19). However, lung metastasis is also very common after various tumors are cured (20, 21). In CRC patients, the assessment of lung metastasis is important for accurate initial staging and subsequent treatment decisions. As we know, Lung metastases grow relatively slowly and have a good prognosis. Surgical resection of lung metastases is considered the best possible treatment (22). Numerous studies have reported that lung metastases resection is associated with favorable 5-year overall survival rates ranging from 24% to 67.8% (23–25). At present, the main difficulties are how to characterize the lung lesions found in the initial stage or postoperative review of CRC patients and how to determine whether they are metastatic or primary lung cancer. Hence, a more accurate, effective and non-invasion prognostic model for ILL with CRC is urgently needed.

Our diagnostic model was built by clinical characteristics and ^18^F-FDG PET/CT features of CRC patients with ILL. Most previous studies have focused on the differential diagnosis of benign and malignant for ILLs with CRC. In our study, The size and SUVmax of lesions were controlled during screening of enrolled patients, which may lead to the diagnosis of more malignant lung lesions. Although this reduces the scope of application of the model, it may improve the diagnostic specificity of the model. Meanwhile, all ILLs in our final analysis were pathologically confirmed, which were also lacking in previous studies.

^18^F-FDG PET/CT is a more accurate, non-invasive method for detecting metastasis of CRC, and has a higher sensitivity than conventional CT in detecting extra-hepatic metastasis (26). ^18^F-FDG PET/CT has an overall accuracy of 90% in diagnosing malignant lung lesions in patients with a history of cancer (27). Our study assessed the clinical value of ^18^F-FDG PET/CT features and Clinical characteristics for distinguishing LM and LC of CRC. Clarification of these characteristics may reduce patient consumption and the use of unnecessary diagnosis, treatment, and follow-up. A diagnostic model using the pleural tags and contour had AUCs of 0.89 and 0.86 in the training and validation sets for distinguishing LM and LC, respectively. The accuracy rate of the subjective evaluation was improved obviously with the assistance of ^18^F-FDG PET/CT diagnostic model for differentiating LM from LC compared to subjective diagnosis alone.

The analysis of nodule morphology may provide important information about the etiology of the lesion. For example, smooth edges and circular are theoretically more indicative of benign lesions or LM, while irregular edges may indicate LC. ILLs with circular contour suggest LM, and ILLs with pleural tags indicate LC in both univariate and multivariate analyses of our study. The results were consist with previous study. Margin characteristics of ILLs were significantly in diagnosis LM with LC in univariate analyses. The “air-bronchogram sign”, which was defined as air within the bronchi or bronchioles passing through airless parenchyma visible as branching linear lucencies (28, 29). The air bronchograms within ILLs was significantly higher in LC than in LM in previous study (4). Perhaps because only 5 ILLs with air-bronchogram, the results significant in univariate analysis, while multivariate analysis was not statistically significant. ILLs contain a GGO component commonly seen in primary lung adenocarcinomas. Only a few reports have described cases of pulmonary metastases (30, 31). Typical LM appear as solid round nodules. In our study, all of GGOs was observed in LC, which was statistically significant in univariate analysis. Thus, GGO density of ILLs may be used to support the diagnosis of LC rather than LM.

The resolution for most PET scanners is 5–6 mm. Therefore, PET is less likely to obtain additional helpful information for smaller lung lesions compared with CT (32, 33). SUV is a semi-quantitative measurement of radiopharmaceutical uptake at the region of interest, which is influenced by many factors (timing of uptake, blood sugar level, capacity effect and statistical noise, etc.). We exclude patients with low SUVmax (<0.5) and small tumor size (<0.8cm) to avoid underestimate of SUVmax. Previous studies have demonstrated the usefulness of ^18^F-FDG PET/CT in the diagnosis of lung metastases for various tumors (32, 34, 35). SUVmax were not found to be significant in our study. The results are consistent with previous reports (8, 27). To further analyze the role of SUVmax, we divided ILLs into higher than mediastinal and lower than mediastinal according to previous literature reports (27). Unfortunately, the SUVmax was not a significant discriminating factor. In addition to the differential diagnosis of ILLS, the capacity of ^18^F-FDG PET/CT to detect extra abdominal disease represents an advantage compared to conventional imaging techniques, allowing better determination of the different M1 categories (36).

The number of ILLs is one of the most common prognostic factors found in previous studies (37, 38). For the pathology was the gold standard in our study, some nodules without pathology were not included in the analysis. As we know, the larger the ILLs, the higher likelihood of malignancy for tumor patients; and that, larger nodules had more distinct morphological features. The diameter of the nodules is less than 1.0 cm, which makes it difficult to perform morphological analysis of such small lesions (39). It has been reported that the failure rate of ^18^F-FDG PET/CT for detecting sub centimeter lung nodules was nearly 50% (40, 41). For these reasons, we did not record and analyze all the nodules per patient.

In addition to ^18^F-FDG PET/CT features, we also analyzed the usefulness of clinical features in differentiating LM from LC. Unfortunately, except for age, other clinical features were not statistically significant in univariate analysis. It may be because the sample size was reduced after we divided patients into training cohort and validation cohort, which affected the statistical results.

Generally, young patients have been considered to have a more aggressive biological behavior and worst prognosis (42, 43). In our study, univariate analysis for age show an statistically significant result in training cohort, which result same with previous study (4). It means that older people are more likely to develop primary lung cancer. Smoking is not only a major risk factor for lung cancer, but also a a risk factor for pulmonary metastasis in CRC patients (44–46). This indicates that patients with CRC who smoke may develop lung cancer or lung metastases. There is no difference was observed in smoking status between LM and LC of CRCs in our study. DFI refers to the time from radical resection of CRC to diagnosis of lung metastasis, which considered a predictor of tumor biology and prognosis (47, 48). However, DFI cannot distinguish LM from LC in our results. This may be due to simultaneous multiple primary carcinoma or lung metastasis in 21 patients. Previous studies have shown that the higher the tumor stage for CRCs, the greater the chance of lung metastasis (49, 50).

Several limitations must be considered in this study. First of all, the retrospective nature of this study may have introduced potential selection and verification biases. Secondly, our sample size was relatively small due to we selected those lesions with histopathological results and larger than 8mm with high uptake for analysis, which limits the accuracy of the conclusions that can be drawn. Third, lymph node dissection was performed only in some patients, so the status of lymph node metastasis was not analyzed as an parameter in our study.

## Conclusion

The pleural tags and contour obtained from ^18^F-FDG PET/CT might be more valuable than other parameters for discriminating LM from LC of CRC. The diagnostic model of ILLs could improve the diagnostic accuracy and contribute to determining an appropriate clinical option for CRCs.

## Data availability statement

The original contributions presented in the study are included in the article/**Supplementary Material**. Further inquiries can be directed to the corresponding authors.

## Ethics statement

As the present analysis has merely a retrospective character, neither Institutional Review Board approval nor informed consent was required by national law.

## Author contributions

RG SY, ZY, NL, and JY were involved in the conception and design of the study. RG, FW, HS and JY were involved in the collection and analysis of data. RG, NL, and JY wrote and revised the manuscript. All authors reviewed and approved the final manuscript. ZY, NL and JY were the principal investigator. All authors provided critical feedback and helped shape the research, analysis, and manuscript, and discussed the results. All authors contributed to the article and approved the submitted version.

## Funding

This work was financially supported by the National Natural Science Foundation (No.81871387, 82171980) and Beijing Natural Science Foundation (No.7202027), Beijing Hospitals Authority Dengfeng Project (DFL20191102).

## Conflict of interest

The authors declare that the research was conducted in the absence of any commercial or financial relationships that could be construed as a potential conflict of interest.

## Publisher’s note

All claims expressed in this article are solely those of the authors and do not necessarily represent those of their affiliated organizations, or those of the publisher, the editors and the reviewers. Any product that may be evaluated in this article, or claim that may be made by its manufacturer, is not guaranteed or endorsed by the publisher.
