# Supplementary material for: A novel diagnostic model for differentiation of lung metastasis from primary lung cancer in patients with colorectal cancer
Source: Front Oncol. 2022 Oct 24;12:1017618. doi: 10.3389/fonc.2022.1017618 (PMC9639374; doi:10.3389/fonc.2022.1017618)
Supplement: Supplementary file 1 [file Table_1.docx]

**Table S1. Comparison of Clinical characteristics of ILLs in training cohort**

| **Characteristic** | LM(n＝39) | LC(n＝21) | *P* value |
| --- | --- | --- | --- |
| Sex (male/female) | 24/15 | 14/7 | 0.694 |
| Age (years) | 59.44±10.39 | 67.95±7.03 | 0.001 |
| History of smoking | 19 | 8 | 0.43 |
| Index tumor location |  |  | 0.312 |
| Right colon | 23 | 11 |  |
| Left colon | 5 | 6 |  |
| Rectum | 11 | 4 |  |
| Index tumor stage |  |  | 0.086 |
| Ⅰ | 6 | 4 |  |
| Ⅱ | 12 | 12 |  |
| Ⅲ | 18 | 5 |  |
| Ⅳ | 3 | 0 |  |
| Extrapulmonary metastasis |  |  | 0.47 |
| Exist | 31 | 19 |  |
| None | 8 | 2 |  |
| Vascular tumor thrombus |  |  | 0.568 |
| Exist | 12 | 5 |  |
| None | 27 | 16 |  |
| CEA |  |  | 0.559 |
| Rise | 16 | 7 |  |
| Normal | 23 | 14 |  |
| DFI | 28.15±33.11 | 24.41±22.28 | 0.828 |

**Table S2. Comparison of ^18^F-FDG-PET/CT features of ILLs in training cohort**

|  | LM(n＝39) | LC(n＝21) | *P* value |
| --- | --- | --- | --- |
| SUVmax | 6.15±3.25 | 7.05±5.80 | 0.92 |
| ≤Mediastinum | 6 | 5 | 0.493 |
| ＞Mediastinum | 33 | 16 |  |
| Size(cm) | 1.71±0.84 | 2.28±1.12 | 0.029 |
| Contour |  |  |  |
| Circular | 18(46.2%) | 1(4.0%) | 0.001 |
| Margin |  |  | <0.05 |
| Smooth | 19(48.7%) | 0(0%) |  |
| Lobulated | 27(69.2%) | 20(95.2%) |  |
| Spiculated | 7(17.9%) | 14(66.7%) |  |
| Air bronchogram | 0(0%) | 3(14.3%) | 0.039 |
| Pleural tags | 13(33.3%) | 18(85.7%) | <0.001 |
| GGO | 0(0%) | 5(23.8%) | 0.004 |
| Location |  |  | ＞0.05 |
| Central | 7(17.9%) | 5(23.8%) |  |
| Peripheral | 32(82.1%) | 16(76.2%) |  |
| Left lung | 17(43.6%) | 11(52.4%) |  |
| Right lung | 22(56.4%) | 10(47.6%) |  |
| Upper lobe | 14(35.9%) | 12(57.1%) |  |
| Middle lobe | 3(7.7%) | 0(0%) |  |
| Lower lobe | 22(56.4%) | 9(42.9%) |  |
| Cavitation | 4(10.3%) | 3(14.3%) | 0.687 |
| Calcification | 0(0%) | 0(0%) |  |

**Table S3. The performance of diagnostic model in training cohort and validation cohort**

| **training cohort** |  | LM | LC | Total | **validation cohort** |  | LM | LC | Total |
| --- | --- | --- | --- | --- | --- | --- | --- | --- | --- |
| D**iagnostic** models | LM | 32 | 2 | 34 | D**iagnostic** models | LM | 30 | 4 | 34 |
|  | LC | 7 | 19 | 26 |  | LC | 8 | 18 | 26 |
|  | Total | 39 | 21 | 60 |  | Total | 38 | 22 | 60 |

**Table S4. The performance of subjective evaluation in training cohort and validation cohort**

| **training cohort** |  | Pathology |  |  | **validation cohort** |  | Pathology |  |  |
| --- | --- | --- | --- | --- | --- | --- | --- | --- | --- |
| R1 |  | LM | LC | Total | R1 |  | LM | LC | Total |
|  | LM | 23 | 4 | 27 |  | LM | 23 | 6 | 30 |
|  | LC | 16 | 17 | 33 |  | LC | 15 | 16 | 30 |
|  | Total | 39 | 21 | 60 |  | Total | 38 | 22 | 60 |
| R2 |  |  |  |  | R2 |  |  |  |  |
|  |  | LM | LC | Total |  |  | LM | LC | Total |
|  | LM | 25 | 5 | 30 |  | LM | 22 | 7 | 29 |
|  | LC | 14 | 16 | 33 |  | LC | 16 | 15 | 31 |
|  | Total | 39 | 21 | 60 |  | Total | 38 | 22 | 60 |
